# A Study on Optimal Machining Conditions and Energy Efficiency in Plasma Assisted Machining of Ti-6Al-4V

**DOI:** 10.3390/ma12162590

**Published:** 2019-08-14

**Authors:** Young-Hun Lee, Choon-Man Lee

**Affiliations:** School of Mechanical Engineering, Changwon National University, 20, Changwondaehak-ro, Uichang-gu, Changwon 51140, Korea

**Keywords:** plasma assisted machining, design of experiment, energy efficiency

## Abstract

This research objective was to determine the significant parameters for effective plasma assisted machining (PAM) of Ti-6Al-4V and to derive optimal processing conditions. PAM parameters such as feed rate, spindle speed, and depth of cut have significant effects on its machining characteristic. In this study, the design of experiments (DOE) was used to select optimal machining conditions for PAM. The signal-to-noise (S/N) ratio was analyzed using the Taguchi method and the contributions of the factors were determined using analysis of variance (ANOVA). Finally, the optimal PAM machining conditions were selected using response optimization. In addition, the energy efficiency of conventional machining (CM) and the PAM were compared. The energy efficiency was analyzed by specific cutting energy. The cutting force and surface roughness of PAM decreased by 60.2% and 70.5%, respectively, in optimal PAM machining conditions.

## 1. Introduction

There has been a sharply increasing demand recently for difficult-to-cut materials in various industrial fields such as medical devices and aerospace. However, because difficult-to-cut materials have exceptional mechanical properties including high strength and abrasion resistance, they are difficult to machine using conventional machining (CM). For example, using CM, tool life and machining quality decrease due to the high cutting force required [1,2,3]. Consequently, many researchers have been actively studying advanced methods for machining difficult-to-cut materials efficiently. One of these, thermally assisted machining (TAM), is attracting attention as an effective method. TAM is a machining method where difficult-to-cut materials are softened by an external heat source prior to engaging the cutting tool. Three types of TAM method have mainly been studied, according to the type of heat source: laser assisted machining (LAM), induction assisted machining (IAM), and plasma assisted machining (PAM). The most active study among these is LAM, where current research and LAM devices have been investigated [4,5]. The development of a control method of a complex tool path and the development of a high-performance tool were proposed as a future research direction. A thermal model was developed to calculate the temperature during the LAM [6,7,8]. Through the developed model, the thermal distribution of the workpiece was obtained and appropriate machining conditions were presented. Numerous research on the machinability of various difficult-to-cut materials have been conducted. Research has been carried out to improve the machinability of Inconel 718, which has high toughness [9,10]. A study was also conducted on the machining of brittle materials such as ceramics and glass [11,12,13,14]. Furthermore, hybrid processes that combine LAM and other processes have been proposed [15,16]. One TAM method, PAM, is more attractive commercially because it is lower in price than the LAM. In addition, since its spot size is larger than that of lasers, PAM has an advantage in the milling process. If an entire section is heated by a small spot, it creates a technical problem because the torch must be rotated at the same rotational speed of the cutting tool. On the other hand, the use of larger spots allows heating including all radial cutting depths, directly in front of the end mill [17].

In PAM, it is very important to study the proper conditions because cutting force and machining quality will vary depending on the machining conditions. PAM has various machining conditions such as feed rate, spindle speed, depth of cut, power, gas flow rate, torch angle, and so on. 

Previous PAM research has been conducted with a focus on machining characteristics. Lopez de Lacalle et al. [17] analyzed tool wear and cutting force using PAM and established that PAM reduced the cutting force by 25% and increased productivity by 350%. Moon and Lee [18] analyzed the cutting force and surface roughness of an AISI 1045 material using PAM and established that PAM reduced the cutting force and surface roughness by 61 to 15% and 79 to 5%, respectively. Kitagawa and Maekawa [19] studied the tool wear and machinability of ceramic materials after applying a plasma heat source to the turning process. Lee et al. [20] studied the machining characteristics according to torch angle, by setting the plasma torch angle as a machining variable.

Studies on the preheating effect of PAM have also been carried out. Kim et al. [21] studied the preheating effect on cylindrical shaped specimens using PAM. Baek et al. [22] studied the preheating effect of laser-plasma heat sources and proposed an appropriate preheating method for effective machining. Lee and Lee [23] studied cutting force by setting the plasma power and gas flow rate as a machining variable and proposed using 2 kW of power and 25 l/min of gas flow rate. Leshock et al. [24] studied heating and operating parameters and systematically analyzed the process control of plasma enhanced machining. They established that PAM reduced the cutting force by 30% and increased tool life by 40%. Wang et al. [25] studied combinations of traditional turning with cryogenically enhanced machining and plasma enhanced machining. Using the proposed machining method, the cutting force and surface roughness were reduced and tool life was extended. They established that PAM reduced the cutting force and surface roughness by 30 to 50% and 250%, and increased tool life by 156%. 

Most of the studies have focused on the aspects of tool life and surface roughness, however, the effect of spindle speed and depth of cut is not well understood. On the other hand, our paper focused on the effect of each factor (feed rate, spindle speed, and depth of cut on cutting force and surface roughness), optimal machining conditions of PAM, and compared CM with PAM through the analysis of energy efficiency. In this paper, the optimal machining conditions for PAM were analyzed by investigating machining characteristics such as cutting force and surface roughness. The selected factors were feed rate, spindle speed, and depth of cut. The experimental optimal machining conditions were determined using the Taguchi method. The influence of factors on the PAM were analyzed using the Taguchi method. Then, the relative characteristics of the factors were determined through analysis of variance. The optimal PAM machining conditions were analyzed using response optimization. In addition, the suitability of the analysis results were evaluated by comparing the results of the prediction equation and the verification experiment. Finally, the energy efficiency of the PAM was analyzed by comparing the specific cutting energy of the CM and the PAM.

## 2. Plasma Assisted Machining

**Figure 1:**
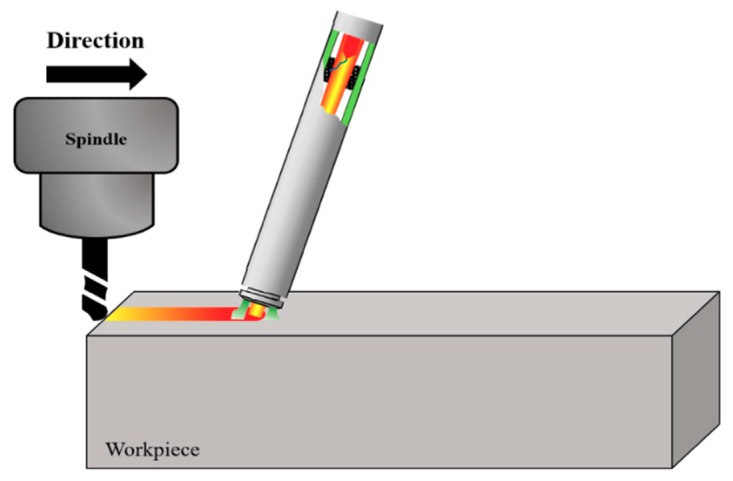
Schematic diagram of the plasma assisted machining (PAM).

PAM is a machining method for difficult-to-cut materials that uses a plasma heat source. Figure 1 shows a schematic design of the PAM. The diameter of the heat source was 6 mm, and the plasma heat source precedes ahead of the cutting tool. The cutting tool then cut the preheated part. The cutting tool used was ∅8 mm, 4 flute, and 70 mm length flat end-mill, in consideration of the size of the plasma heat source.

**Figure 2:**
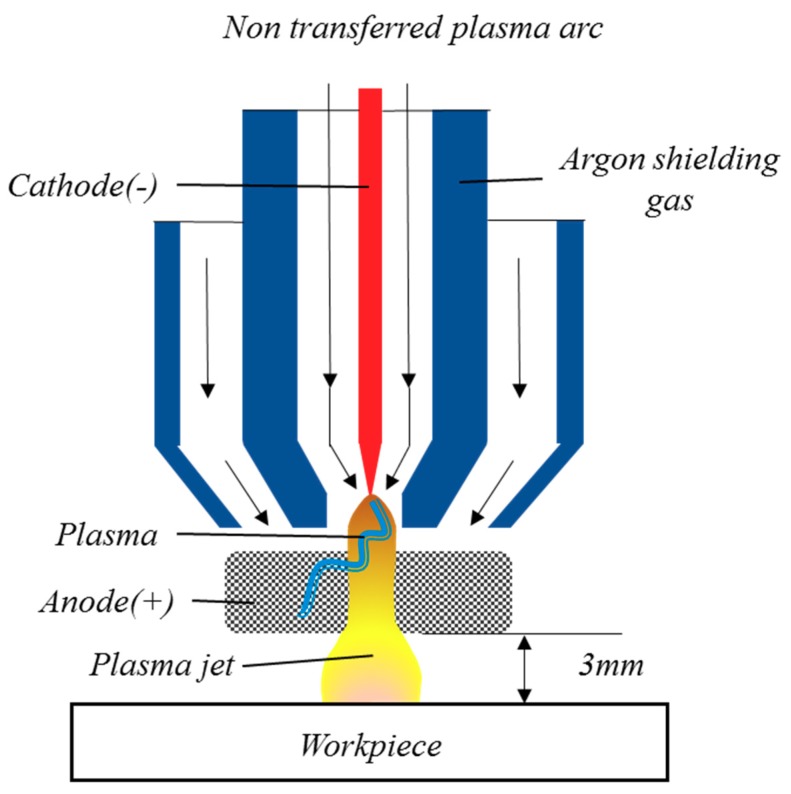
Design of a non-transfer plasma torch.

The plasma torch was divided into a non-transfer torch and a transfer torch, according to the generation method. The non-transfer torch generates plasma inside the torch, while the transfer torch generates plasma between the workpiece and the torch [18,19]. In this study, the non-transfer torch was used. Figure 2 shows the design of a non-transfer torch.

## 3. Finite Element Method

### 3.1. Thermal Analysis

**Figure 3:**
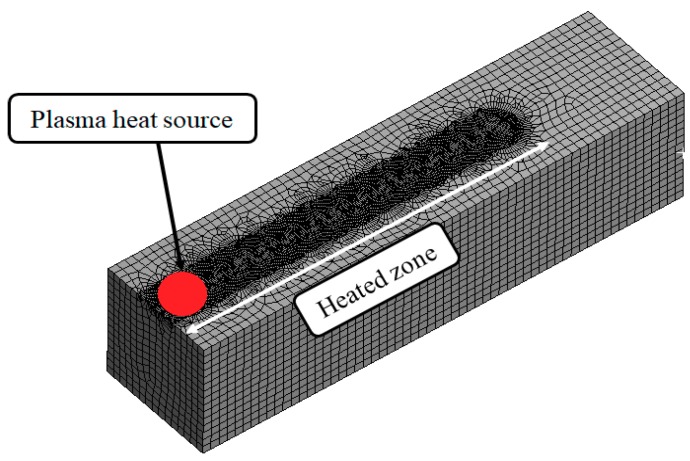
Analysis model.

The material used was Ti-6Al-4V Alpha-Beta, which is widely employed in aviation and automobile parts. First, a plasma heating analysis of the titanium was performed. The thermal analysis was required to select the effective depth of cut. The preheating temperature was selected considering the tensile strength of Ti-6Al-4V, according to temperature. The thermal analysis was undertaken using the ANSYS work-bench where a transient thermal analysis was performed. Figure 3 shows the thermal analysis model used in this study. The material size was 15 mm x 15 mm x 60 mm (Thickness × Width × Length). The mesh was applied using the hex dominant method for the thermal analysis. The mesh size of the heated zone was divided by 0.5 mm for accurate analysis, and the other parts were divided by 1 mm. The diameter of the plasma heat source was 6 mm. The number of nodes and elements were 69,681 and 203,045.

The heat transfer equations and boundary conditions used in the plasma analysis are shown in Equations (1) and (2) [23,26].

(1) $$\frac{\partial }{\partial_{x}}\left(k\frac{\partial T}{\partial x}\right)+\frac{\partial }{\partial y}\left(k\frac{\partial T}{\partial y}\right)+\frac{\partial }{\partial z}\left(k\frac{\partial T}{\partial z}\right)+\dot{Q}=\rho C_{\rho }\frac{\partial T}{\partial t}$$

In Equation (1), $T,\;t,\;k,\;C_{\rho },\;\rho ,$ and $\dot{Q}$ represent the temperature, time, thermal conductivity, specific heat, density, and heat generation rate, respectively.

(2) $$-k\frac{\partial T}{\partial z}=q\left(x,y\right)-h\left(T-T_{O}\right)$$

In Equation (2), q and h represent the heat flux and heat transfer coefficient.

### 3.2. Results of Analysis

**Figure 4:**
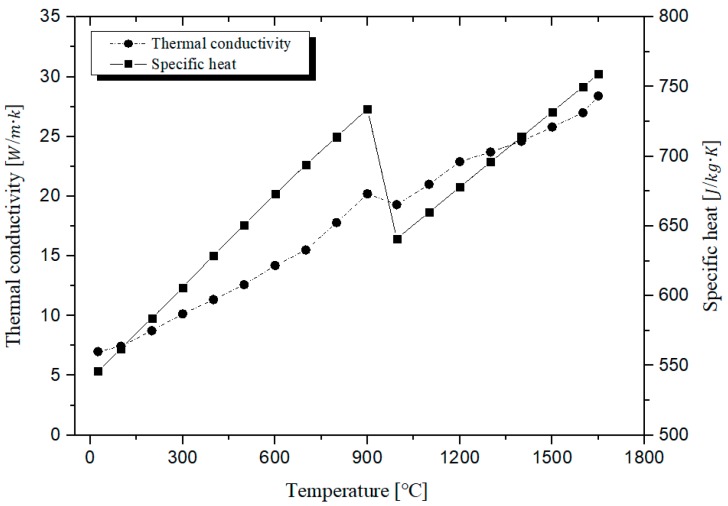
Thermal conductivity and specific heat of Ti-6Al-4V [27,28].

**Figure 5:**
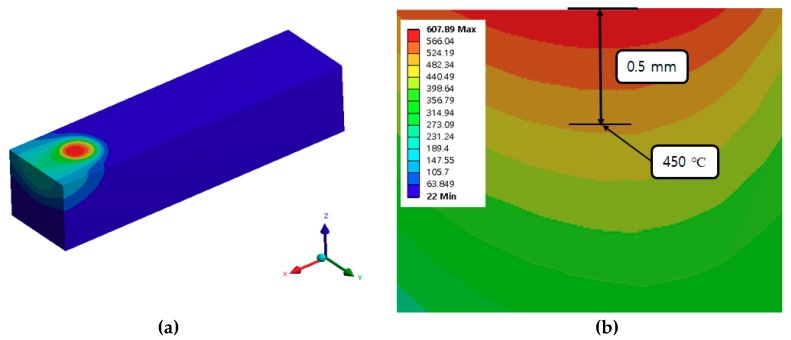
The thermal analysis results of a preheating temperature of 600 °C and a feed rate of 250 mm/min, (**a**) The temperature distribution of workpiece; (**b**) Section view in the x-z plane.

Table 1 shows the chemical composition of Ti-6Al-4V. The heat flow conditions were applied to the plasma heat source. The convection condition of workpiece was applied to $5\;W/m^{2}$·°C, which was equal to the air condition. The density of Ti-6Al-4V was applied to 4.5 $g/{\mathrm{cm}}^{3}$. The specific heat and thermal conductivities of the Ti-6Al-4V are shown in Figure 4. The tensile strength of Ti-6Al-4V decreased rapidly by 49% to 64% when compared to the strength of the room temperature, in the range of about 450 to 600 °C [27,28]. The preheating temperature was selected considering the tensile strength of Ti-6Al-4V, according to temperature. Therefore, the preheating temperature on the surface was set to 600 °C and depth of cut was set to 0.5 mm, considering the tensile strength which sharply decreased at 450 °C. In this thermal analysis, the feed rate was set to 250 mm/min, the fastest among the feed rate levels. Since the heat effect was lowest at a feed rate of 250 mm/min, the thermal analysis was performed at a feed rate of 250 mm/min to ensure the fairness of the experiment. Figure 5 shows the temperature distribution and depth of cut for a preheating temperature of 600 °C and a feed rate of 250 mm/min. As a result of the thermal analysis, the depth of cut was set to be about 0.5 mm.

## 4. Experiment

### 4.1. Experiment Setup

**Figure 6:**
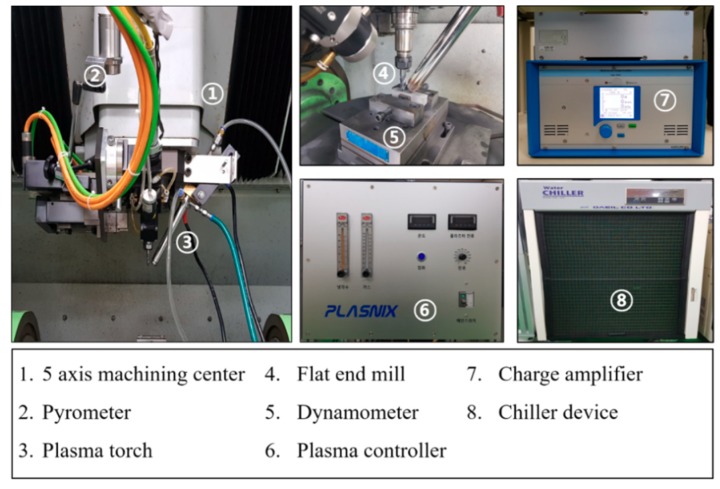
Experimental setup.

Figure 6 shows the experimental setup used in the PAM. In this experiment, the machining experiment was performed using a 5-axis machining center (Hyundai WIA, Hi-V560M, Changwon, Korea) and the torch (PLASNIX, IGBT, Incheon, Korea) used to generate the plasma was mounted on the right side of the spindle. The plasma torch was a non-transfer type torch and argon gas was used. In addition, a coolant device was installed to prevent the plasma torch from being damaged by heat at a high temperature. A dynamometer (Kistler, 9257B, Winterthur, Switzerland) was installed to measure the cutting force. The preheating temperature of the material surface was measured with a pyrometer (Dr. Mergenthaler GmbH & Co. KG, LPC03, Neu-Ulm, Germany). The workpiece used was Ti-6Al-4V and the cutting tool used was a flat-end mill (WIDIN, ZE324081, Changwon, Korea). Additionally, the surface roughness of the workpiece was analyzed using a surface roughness tester (KOSAKA, SEF-3500K, Tokyo, Japan). 

### 4.2. Design of Experiments

In PAM, machining characteristics are affected by various factors. In order to quantitatively understand the influence of the factors on the machining characteristics, the experiments should be carried out after considering all combinations of factors. However, to do so would increase the number of experiments and with high cost and time consumption. Therefore, a small number of experiments were performed using the Taguchi method. The robust design of the Taguchi method allows one to find the large influential factors to control, and minimizes the influence of noise by maximizing the influence of these factors. The Taguchi method uses the signal to noise ratio (S/N ratio) to evaluate the fluctuation in product quality in the parameter design [28,29,30,31,32,33,34]. 

In this experiment, the Taguchi method was applied using MINITAB. The PAM evaluation characteristics were analyzed in terms of cutting force and surface roughness. The cutting force and surface roughness were calculated by using the smaller-the-better characteristic. The experiments were performed using orthogonal arrays by setting controllable machining conditions factors. The signal-to-noise ratio according to the quality characteristic was calculated and a factor indicating the maximum S/N ratio was found. The smaller characteristic is shown in Equation (3).

(3) $$S/N=-10\log\left[\frac{1}{n}\overset{n}{\underset{i=1}{\sum }}y_{i}^{2}\right]$$

where $y_{i}$ represents the data obtained from the experiment, and n represents the number of experiments.

### 4.3. Machining Condition

The signal-to-noise ratio according to the quality characteristic was calculated and the factor indicating the maximum S/N ratio was found. The feed rate, spindle speed, and depth of cut were set as the control factors in this study. The feed rate levels were set at 50 mm/min, 150 mm/min, and 250 mm/min and the spindle speed levels were set at 2000 rpm, 7000 rpm, and 12,000 rpm.

When using a general cutting tool recommended by WIDIN Co. Ltd for Ti-6Al-4V, it is normally used at a feed rate of 50 mm/min and a spindle speed of 2000 rpm. In order to experiment with high speed machining to improve productivity efficiency, the feed rate and spindle speed were set at 250 mm/min and 12,000 rpm. The levels of the depth of cut were determined by dividing the effective depth of cut into three parts based on the thermal analysis result. The gas flow rate and the torch angle, which are fixed parameters, were defined using the optimal values obtained in the previous study [23]. Table 2 shows the PAM machining conditions. The three factors and the three levels were applied in this experiment. Table 3 shows the $L_{9}\left(3^{4}\right)$ table of orthogonal arrays.

### 4.4. Measurement

**Figure 7:**
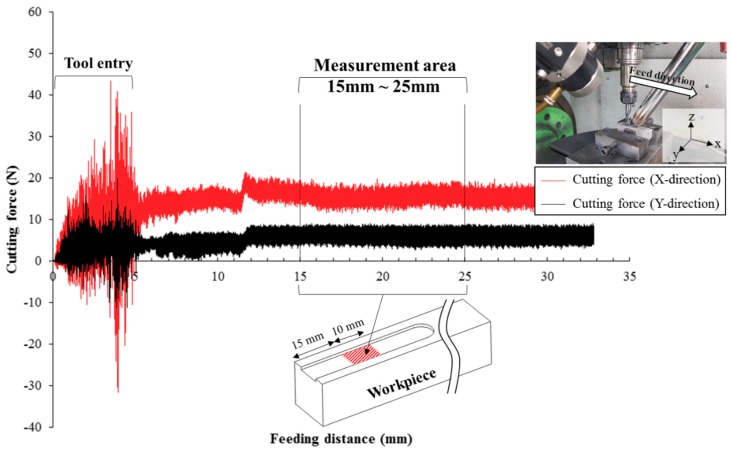
Measurement area of the cutting force and surface roughness.

Cutting force and surface roughness were measured as shown as Figure 7. X (feed direction) and Y (normal direction) axis cutting force was measured within 10~20 s considering the entry of the tool and was calculated by Equation (4).

(4) $$\mathrm{Mean}\;\mathrm{of}\;\mathrm{resultant}\;\mathrm{cutting}\;\mathrm{force}=\frac{1}{n}\overset{n}{\underset{k=1}{\sum }}\sqrt{F_{xk}^{2}+F_{yk}^{2}}$$

where n is the number of time intervals equal to 10,000.

$F_{x}$ is a feed direction force and $F_{y}$ is a normal direction force. The confidence interval was obtained by calculating the standard deviation between the measured cutting forces and average cutting force by three repeated experiments. The surface roughness was also measured three times.

## 5. Results and Discussion

### 5.1. Main Effect 

**Figure 8:**
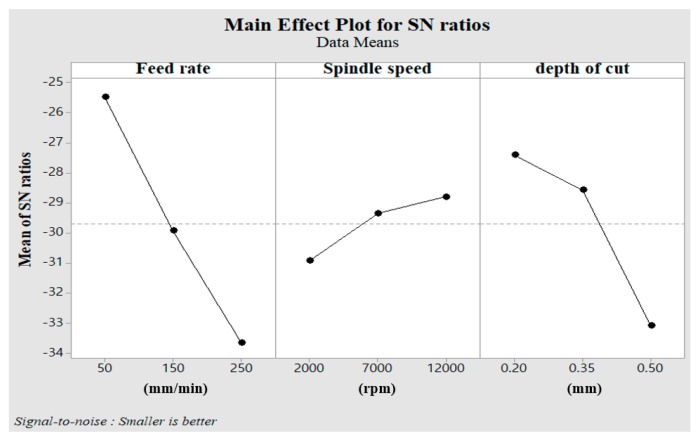
Main effect of the S/N ratio on cutting force.

**Figure 9:**
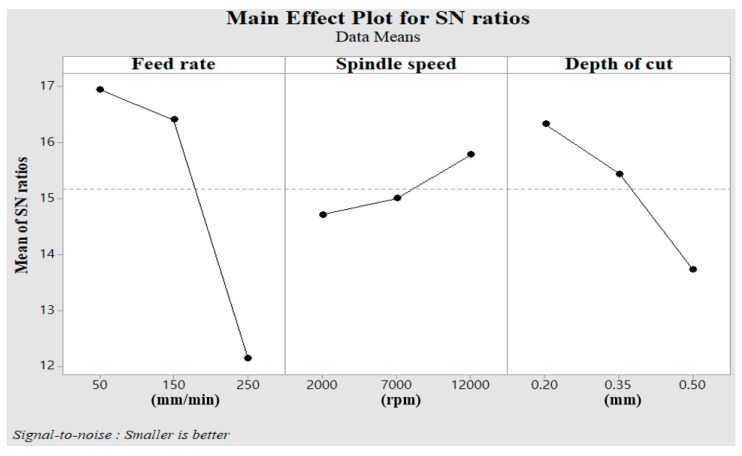
Main effect of the S/N ratio on surface roughness.

As indicated in the designated orthogonal array table, nine different PAM experiments were conducted. Table 4 shows the results, obtained by calculating the average values of the experiments. Figure 8 shows the main effect of the S/N ratio on the cutting force. The factor that had the greatest influence on the cutting force was feed rate, and the next was depth of cut and spindle speed, as shown in Table 5. Figure 9 shows the main effect of the S/N ratio on surface roughness. The factor that had the greatest influence on surface roughness was feed rate, and the next was depth of cut and spindle speed, as seen in Table 6.

The lowest cutting force was confirmed at feed rate A and level 1 (A1), the spindle speed B and level 3 (B3), and the depth of cut C and level 1 (C1) were used. The lowest surface roughness was confirmed when feed rate A and level 1 (A1), spindle speed B and level 3 (B3), and depth of cut C and level (C1) were used.

### 5.2. Analysis of Variance

An analysis of variance (ANOVA) was conducted to confirm the relative characteristics of the factors. Table 7 and Table 8 show the results of the ANOVA regarding cutting force and surface roughness. In the results, feed rate was the most influential factor on cutting force. The contribution of factors to the cutting force were approximately 54.15% for the feed rate, 7.9% for the spindle speed, and 36.79% for the depth of cut. Similarly, the feed rate was the most influential factor in the surface roughness. The contribution of factors to the surface roughness were approximately 77.24% for the feed rate, 4.12% for the spindle speed, and 16.25% for the depth of cut.

### 5.3. Response Optimization

A response optimization analysis was performed using the results of the Taguchi method. The response optimization helps to distinguish the combinations of factors that optimize a series of responses in common. This is helpful when considering the effect of several factors on the response. Table 9 and Table 10 show the response optimization and the results of the response optimization. In the results, the feed rate, the spindle speed and the depth of cut were analyzed at 50 mm/min, 12,000 rpm, and 0.2 mm. Desirability was confirmed at 1.

### 5.4. Verification Experiment of and Prediction Equations

Next, verification experiments were conducted to evaluate the suitability of the analysis results. The prediction equations of the cutting force and the surface roughness are shown in Equations (5) and (6).

(5) $$\begin{array}{ll} F_{C}=34.50- & 14.98\left(F50\right)-0.58\left(F150\right)+15.55\left(F250\right)+6.24\left(S2000\right)\\ & -0.92\left(S7000\right)-5.32\left(S12000\right)-9.61\left(D0.2\right)\\ & -4.64\left(D0.35\right)+14.25\left(D0.5\right) \end{array}$$

(6) $$\begin{array}{ll} F_{S}=0.182- & 0.038\left(F50\right)-0.028\left(F150\right)+0.066\left(F250\right)\\ & +0.012\left(S2000\right)-0.001\left(S7000\right)-0.014\left(S12000\right)\\ & -0.02\left(D0.2\right)-0.009\left(D0.35\right)+0.03\left(D0.5\right) \end{array}$$

where $F_{C}$ represents the cutting force, while $F_{S}$ represents the surface roughness.

**Figure 10:**
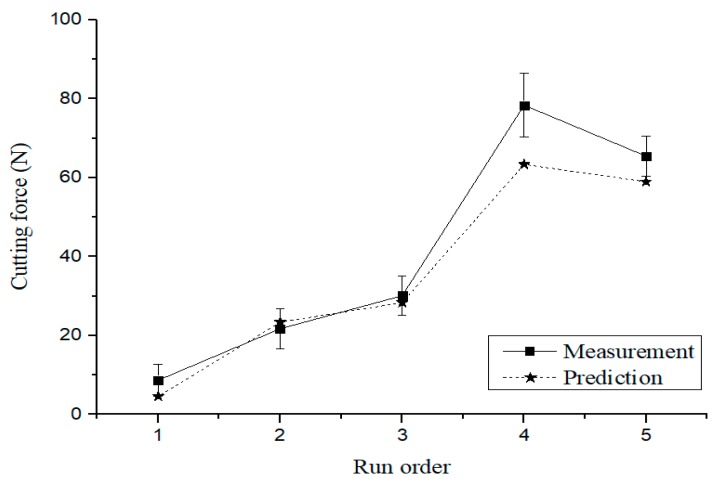
Comparison of the results of the prediction equation and the verification experiments for the cutting force.

**Figure 11:**
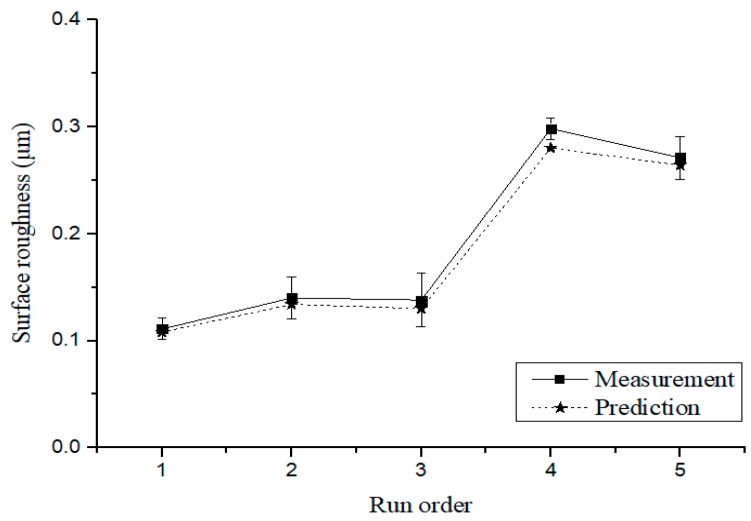
Comparison of the results of the prediction equation and the verification experiments for the surface roughness.

Table 11 show the machining conditions of the verification experiments. The five experiments were conducted by randomly adding the levels of the factors including the optimal machining conditions (Exp. No. 1), as specified in Table 11. Figure 10 compares the results of the prediction equation and the verification experiments for cutting force. The maximum error rate was verified as approximately 19.1%. Figure 11 shows a comparison of the results of the prediction equation and the verification experiments for surface roughness. The maximum error rate was approximately 6.04%.

### 5.5. Specific Cutting Energy

The specific cutting energy is the energy required to cut a unit volume of material per unit time. In this research, the specific cutting energy was used to verify the energy efficiency of the CM and PAM. The specific cutting energy, U, is defined as [28]

(7) $$U=\frac{F_{t}\times V}{MRR}$$

In Equation (7), $F_{t},\;V$, and MRR represent the cutting force, the cutting speed, and the material removal rate, respectively. The material removal rate, MRR, is defined as [28]

(8) MRR=F×W×D

In Equation (8), F, W, and D, represent the feed rate, the width of cut, and the depth of cut.

**Figure 12:**
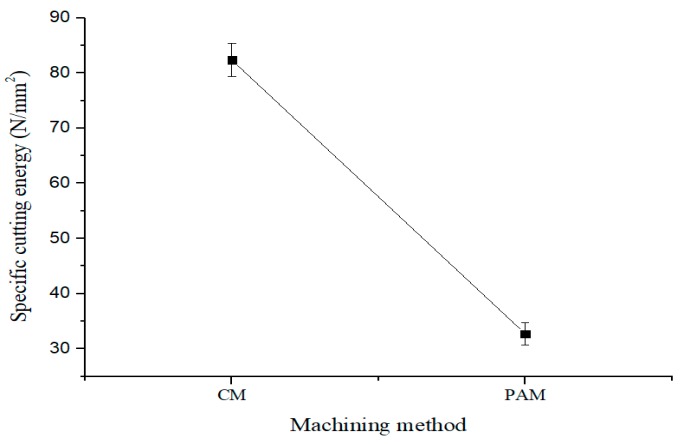
Result of specific cutting energy in the CM and PAM.

Figure 12 shows the results for specific cutting energy in the CM and PAM. The specific cutting energy for PAM decreased by approximately 60.2% compared to the CM. Therefore, it can be seen that PAM increased the specific cutting energy efficiency.

## 6. Conclusions

In this study, the optimal machining conditions for plasma assisted machining of Ti-6Al-4V materials were studied. Thermal analysis was performed to select the effective depth of cut of Ti-6Al-4V. The optimal machining conditions for PAM were determined by DOE, and the evaluation characteristics were analyzed by cutting force and surface roughness. The prediction equations for cutting force and surface roughness were obtained using regression analysis.

The conclusions of this study are as follows.

(1) The preheating temperature and the depth of cut were determined as 600 °C and 0.5 mm, respectively. These values were determined by thermal analysis and considered the tensile strength change of Ti-6Al-4V by temperature.
(2) The relative contributions of the factors were analyzed through the analysis of variance. Feed rate had the most influence on cutting force and surface roughness.
(3) Prediction equation was verified by verification experiments. The optimal machining conditions for PAM were determined as a feed rate of 50 mm/min, spindle speed of 12,000 rpm, and depth of cut of 0.2 mm.
(4) In optimal machining conditions, the cutting force of CM and PAM was 21.84 N and 8.68 N, respectively. The cutting force of PAM was decreased by 60.2% when compared to CM. In addition, the surface roughness of CM and PAM was 0.376 um and 0.111 um, respectively. The surface roughness of PAM was improved by 70.47% when compared to CM.
(5) The energy efficiency of PAM was analyzed by comparing the specific cutting energy. The specific cutting energy of PAM was 32.9 $N/{\mathrm{mm}}^{2}$. The energy efficiency of PAM improved to above 60.26% when compared to CM.

## Figures and Tables

**Table 1:** Chemical composition of Ti-6Al-4V (wt.%).

Ti	Al	V	Fe	O	C	N	H
88.02%	6.75%	4.5%	0.4%	0.2%	0.08%	0.03%	0.01%

**Table 2:** Machining conditions.

Factors	Code	Level		
		1	2	3
Feed rate (mm/min)	A	50	150	250
Spindle speed (rpm)	B	2000	7000	12,000
Depth of cut (mm)	C	0.2	0.35	0.5
Constant factors	Gas flow rate: 25 l/min Torch angle: 60° Preheating temperature: 600 ℃			

**Table 3:** $L_{9}\left(3^{4}\right)$ Table of orthogonal arrays.

Run Order	Feed Rate (A)(mm/min)	Spindle Speed (B)(rpm)	Depth of Cut (C)(mm)
1	1	1	1
2	1	2	2
3	1	3	3
4	2	1	2
5	2	2	3
6	2	3	1
7	3	1	3
8	3	2	1
9	3	3	2

**Table 4:** $L_{9}\left(3^{4}\right)$ Experimental results of the cutting force and surface roughness.

Exp. No.	Feed Rate (mm/min)	Spindle Speed (rpm)	Depth of Cut (mm)	Cutting Force (N)	Surface Roughness (μm)
1	50	2000	0.2	18.548	0.128
2	50	7000	0.35	13.61	0.131
3	50	12,000	0.5	26.418	0.171
4	150	2000	0.35	33.481	0.169
5	150	7000	0.5	49.648	0.178
6	150	12,000	0.2	17.148	0.115
7	250	2000	0.5	70.188	0.287
8	250	7000	0.2	37.485	0.241
9	250	12,000	0.35	42.484	0.218

**Table 5:** Response table mean S/N ratio for the cutting force, according to the machining conditions.

Level	Feed Rate (mm/min)	Spindle Speed (rpm)	Depth of Cut (mm)
1	−25.49	−30.93	−27.42
2	−29.94	−29.36	−28.58
3	−33.66	−28.81	−33.09
Delta	8.16	2.12	5.68
Rank	1	3	2

**Table 6:** Response table mean S/N ratio for the surface roughness, according to the machining conditions.

Level	Feed Rate (mm/min)	Spindle Speed (rpm)	Depth of Cut (mm)
1	16.95	14.71	16.33
2	16.41	15.00	15.44
3	12.14	15.79	13.72
Delta	4.81	1.07	2.61
Rank	1	3	2

**Table 7:** Analysis of variance for the cutting force.

Factors	Degree of Freedom	Sum of Squares	Mean of Squares	F Ratio	Contribution (%)
Feed rate	2	1399.34	699.67	699.67	54.15
Spindle speed	2	204.11	102.05	102.05	7.9
Depth of cut	2	950.78	475.39	475.39	36.79
Error	2	30.20	15.01		1.16
Total	8	2584.25			100.00

**Table 8:** Analysis of variance for the surface roughness.

Factors	Degree of freedom	Sum of Squares	Mean of Squares	F ratio	Contribution (%)
Feed rate	2	0.02	0.01	32.22	77.24
Spindle speed	2	0.001	0.0005	1.72	4.12
Depth of cut	2	0.004	0.002	6.78	16.25
Error	2	0.0006	0.0003		2.4
Total	8	0.026			100.00

**Table 9:** Response optimization.

Parameter	Goal	Target	Upper	Weight	Importance
Cutting force	Minimum	13.610 N	70.188 N	1	1
Surface roughness	Minimum	0.115 μm	0.287 μm	1	1

**Table 10:** Result of the response optimization.

Feed Rate (mm/min)	Spindle Speed (rpm)	Depth of Cut (mm)	Cutting Force Optimization Plot (N)	Surface Roughness Optimization Plot (μm)	Desirability
50	12,000	0.2	4.6	0.108	1

**Table 11:** Machining conditions of the verification experiments.

Exp. No.	Feed Rate (mm/min)	Spindle Speed (rpm)	Depth of Cut (mm)
1	50	12,000	0.2
2	150	7000	0.2
3	150	12,000	0.35
4	250	7000	0.5
5	250	12,000	0.5
